# A worksite intervention to reduce the cardiovascular risk: proposal of a study design easy to integrate within Italian organization of occupational health surveillance

**DOI:** 10.1186/s12889-015-1375-4

**Published:** 2015-01-21

**Authors:** Giuseppe Mastrangelo, Gianluca Marangi, Danilo Bontadi, Emanuela Fadda, Luca Cegolon, Melania Bortolotto, Ugo Fedeli, Luciano Marchiori

**Affiliations:** Department of Cardiac Thoracic and Vascular Sciences, University of Padua, Via Giustiniani 2, 35128 Padua, Italy; Health and Safety at Work Department (SPSAL), Local Health Authority No.20, Veneto Region, Via Salvo D’Acquisto 7, 37122 Verona, Italy; National Association of Occupational Physicians (Associazione Nazionale dei Medici D’Azienda e Competenti, ANMA Veneto), Via Nazareth 2, 35128 Padova, Italy; Department of Philosophy, Sociology, Education and Applied Psychology, University of Padua, Piazza Capitaniato 3, I-35139 Padova, Italy; Regional Epidemiology Service, Veneto Region, Passaggio Luigi Gaudenzio 1, 35131 Padua, Italy

**Keywords:** Worksite health promotion, Cardiovascular disease, Cardiovascular risk, Pretest-posttest design

## Abstract

**Background:**

Despite the substantial amount of knowledge on effectiveness of worksite health promotion (WHP) in reducing cardiovascular disease (CVD) risk, WHP programs are not systematically applied in Italy. The aim was to design an intervention easy to integrate within the Italian organization of workplace health surveillance.

**Methods:**

We used the “pretest-posttest design”. Workers were employed in multiple occupations and resident in Veneto region, Italy. Occupational physicians (OPs) performed all examinations, including laboratory evaluation (capillary blood sampling and measure of glycaemia and cholesterolemia with portable devices), during the normal health surveillance at worksite. CVD risk was computed based on sex, age, smoking habit, diabetes, systolic pressure and cholesterol level. After excluding those with <40 years of age, missing consent, CVD diagnosis or current therapy for CVD, missing information, CVD risk <5%, out of 5,536 workers 451 underwent the intervention and 323 male workers were re-examined at 1 year. CVD risk was the most compelling argument for changing lifestyle. The counseling was based on the individual risk factors. Individuals examined at posttest were a small fraction of the whole (6% = 323/5,536). In these workers we computed the ratio pretest/posttest of proportions (such as percent of subjects with cardiovascular risk >5%) as well as the exact McNemar significance probability or the exact test of table symmetry.

**Results:**

CVD risk decreased by 24% (McNemar p = 0.0000) after the intervention; in a sensitivity analysis assuming that all subjects lost to follow-up kept their pretest cardiovascular risk value, the effect (−18%) was still significant (symmetry p < 0.0000). Each prevented CVD case was expected to cost about 5,700 euro.

**Conclusions:**

The present worksite intervention promoted favorable changes of CVD risk that were reasonably priced and consistent across multiple occupations.

## Background

The worksite has been proposed by the World Health Organization (WHO) as a priority setting for health promotion in the 21^st^ century: *The worksite directly influences the physical, mental, economic and social well-being of workers and in turn the health of their families, communities and society. It offers an ideal setting and infrastructure to support the promotion of health of a large audience* [1].

Worksite health promotion programs originated in US from executive fitness programs that were created in the years after World War II. Initiated by business leaders who endorsed the benefits of a healthful lifestyle, the number of in-house corporate programs grew steadily throughout the 1970s. During the next decades, employer benefits began to focus on management of prevalent chronic conditions (obesity, diabetes, heart disease, cancer, and depression) instead of focusing on fitness and were increasingly offered to employees of all job levels [2]. Work health programs (WHPs) carried out in the past decade showed, in particular, promising results in contrasting the modifiable risk factors of cardiovascular diseases (CVDs) defined as: 1) physical inactivity, 2) tobacco use, 3) hypertension, 4) dyslipidemia, 5) poor diet, 6) hyperglycemia, and 7) elevated psychological stress [3]. Several review papers have been recently issued on WHPs and cardiac rehabilitation, authored by leading authors from the United States [4], Canada [5], Brazil [6], Europe [7], India [8] and Japan [9]. Although the delivery models, level of development/utilization, and legislative support varied among countries, these reviews clearly indicated that worksite health and wellness are important lifestyle intervention strategies and should be viewed as integral components of global healthcare with respect to combating CVDs [3].

Little has been done in Italy. Even the specific notion of WHP and lifestyle modification interventions is unknown within the Italian legal system, particularly in the recent set of rules for health and safety in workplaces contained in Legislative Decree 9/4/2008 No. 81 (updated in 2013) [10]. A barrier is probably the fact that in Italy common diseases are entrusted to the general practitioner (public service), while occupational diseases are assigned to the occupational physician (private service). Employers are reluctant to support additional financial costs to improve their employees’ health, because this task falls to public health care organizations. On the other hand, employed individuals are unable to attend primary care services during the working day and may not wish to utilize their “citizen time” (time spent outside work) for this; in addition, males are less likely than their female counterparts to schedule annual health checks, seek medical advice, or attend educational meetings [11].

Despite the substantial amount of knowledge on effectiveness of WHPs, these interventions are not systematically applied in Italy. We therefore aimed to design an educational intervention that could be easy to integrate within the Italian current organization of occupational health surveillance and reasonably priced.

## Methods

### Study design

Quasi-experimental study designs, often described as pre-post intervention studies or before-and-after studies, are common in the medical literature. We used a quasi-experimental design and precisely the “one-group pretest-posttest design” [12].

The key outcome was reducing the cardiovascular risk over the next 10 years. The latter was computed with an algorithm − proposed by the European Society of Cardiology [13] and acknowledged by the Istituto Superiore di Sanità (Italian National Institute of Health) [14], combining information on sex, age, smoking habit, diabetes, blood pressure (mmHg), and blood cholesterol level (mg/dl). The modifiable risk factors (smoking, blood pressure, cholesterol), along with physical inactivity and alcohol intake were targeted by the intervention. There are multiple primary outcomes and this is, therefore, a multi-faceted worksite intervention to promote favorable changes in cardiovascular disease risk factors.

Study size was set at about 5,000 workers based on the available budget rather than power considerations. Workers were employed in a wide range of occupational sectors (private and public businesses, industry and services) and were resident in various provinces (Padova, Verona, Vicenza) of Veneto, Northeastern region of Italy. An intervention across multiple occupational groups from separate geographic communities could increase confidence that the intervention was responsible for a change in outcome. The ultimate rationale of this procedure was, therefore, assessing consistency of results.

Lastly, an unstructured interview (qualitative method) was conducted by a trained occupational physician (OP) among a small group of information-rich workers to answer the questions: «How did the intervention have that effect?»; and «What was the reaction of participants to the intervention?» [15].

### Sampling frame and running the study

A snowball sampling method was used to select these workers. Two authors (LM and GM) chose the occupational physicians based on their scientific interests; and OPs chose the companies where they had the best relationships with both employers and employees. An invitation letter was sent to the management of these companies, explaining the aim and methods of the study; the companies willing to cooperate constituted the final sample of 5,536 workers.

Before investigation, OPs were trained on counseling techniques, mainly focused on diagnosing the worker’s motivational state to change risky behaviors. Each OP was given a fixed incentive (20 euro) for each worker examined.

All investigations were performed by OPs during the normal health surveillance and took place in the worksite. A computer aided interviewing software (Microsoft Access) was set up to store the data. Information was collected on: lifestyle factors (physical activity, cigarette smoking, alcohol consumption); past medical history (particularly, occurrence of CVDs, diabetes and obesity) and whether the subject was under therapy for diabetes, hypertension and hypercholesterolemia. At physical examination, blood pressure was measured twice at 4–5 minutes distance, always right arm and worker in upright position; the lowest value was used in the statistical analysis. The laboratory evaluation was performed in the workplace itself, collecting specimens of capillary blood and measuring glycaemia and cholesterolemia with portable devices. The procedure for collecting capillary blood specimens by fingerstick was that recommended by Centers for Disease Control [16]. The cardiovascular risk over the next 10 years (CVD risk) was computed with the above algorithm and scored in classes (<5%; 5-10%; 10-15%; 15-20%; 20-30%; >30%). After 12 months, workers were re-examined with the same protocol.

The exams began after a letter of information to the supervisory body for workplace safety and health of the relevant Local Health Authority. The project was run from January 2011 up to December 2012. Clearance by Ethics Committee was not necessary because the study was a mandatory activity deliberated by Veneto Region with a formal act (Regional Decree n. 2008 3 Aug 2010). All workers signed an informed consent at enrollment.

The original 5,536 workers were divided in several subsets during the course of the study. We did not consider 2,062 subjects aged less than 40 years, 65 individuals not giving the consent, 36 patients already affected by CVD, and 537 under current therapy for hypertension, hypercholesterolemia and diabetes. 2,836 subjects older than 40 years without past CVD history or current therapy for diabetes, hypertension, and hypercholesterolemia underwent laboratory evaluation. 59 workers with missing information on one or more of the six components (sex, age, smoking, diabetes, blood pressure and cholesterol) used to estimate the cardiovascular risk, and 2,326 workers with a CVD risk below 5% were excluded. The remaining 451 underwent the educational intervention. Out of the latter, 330 workers (323 males and 7 females) with a CVD risk >5% were re-examined at 1 year, while 121 (26.8% = 121/451) were lost to follow-up. All analyses were carried out in the 323 males because seven subjects could not be used to arrive at any conclusions regarding female gender.

### Educational intervention

There were two aspects: motivation and education. The most compelling argument for changing lifestyle was the estimated risk of CVDs in the next 10 years. Then subjects received an individualized counseling based on the presence of risk factors. Physical activity was generally mistaken with “exercise” (activity that is planned, structured, repetitive, and purposeful). In agreement with World Health Organization [17], workers were recommended to do at least 150 minutes of moderate-intensity aerobic physical activity throughout the week (example 30 minutes 5 times/week). For diet, recommendations were to limit energy intake from total fats and shift from saturated fats to unsaturated fats, increase consumption of fruits, vegetables, legumes, whole grains and nuts, limit the intake of free sugars and limit salt consumption from all sources [17]. Subjects with hypertension and/or hypercholesterolemia or hyperglycemia were interviewed about their attitude towards lifestyle change; whenever they could not cope to recommendations they were referred to their general practitioners for medical therapy, even when the CVD risk was lower than 20%. Likewise, most smokers with CVD risk >5% were addressed to receive an anti-smoking counseling from counselors with educational competence. In other words, we used an aggressive approach that combined both a primary and secondary prevention.

### Statistical analysis

In order to determine whether the intervention had the intended effect we calculated proportions with the factor (such as, proportions of smokers, before and after), the ratio between proportions (point estimates and confidence intervals) and the exact McNemar significance probability (for 2 × 2 tables). For outcome variables with multiple discrete levels (k × k tables), we performed an exact test of table symmetry.

A sensitivity analysis was carried out, performing an exact test of table symmetry on 451 subjects that included 121 subjects (26.8%) lost to follow-up. The latter contributed to the analysis assuming that their pretest value of cardiovascular risk remained unchanged at 1 year.

We coded a binary variable (delta) that was 1 if pretest CVD risk was higher that posttest CVD risk, and 0 otherwise. A low value of delta seemingly indicates a worst impact of the intervention. Since figures became too sparse in the subset of 330 workers undergoing intervention, occupational categories were merged in four groups: “basic metals” (original category); “other industries” (multiple categories); hospital workers (original category); other service workers (multiple categories). Using delta as outcome we fitted two models of logistic regression where the predictors were age, gender and work sectors (model 1); or age, gender, posttest smoking, posttest blood cholesterol, posttest systolic blood pressure and work sectors (model 2). In all models the work sector with the lowest value of delta was the reference. Odds ratios (OR) with 95% confidence interval (CI) and p-value were calculated with Stata 13 (Stata Corporation, College Station, Texas, USA).

A statistical process [13] had determined a detectable characteristic (CVD risk) associated with an increased chance of experiencing future unwanted outcomes. By identifying risk factors before the occurrence of the event, we developed targeted interventions to mitigate their impact. In order to obtain the prevented cases of CVD expected by the intervention, we multiplied in each class of risk the median CVD risk by the number of subjects. The sum of the latter values were the cases expected at pretest (A) or posttest (B). The number of CVD cases potentially prevented by the intervention was the difference (A – B). The cost outcome analysis was obtained by dividing overall cost by the number of potentially prevented cases.

## Results

Table 1 shows in each occupational category the number of people, percent of males, mean and standard deviation of age, separately in the whole population and in the intervention group. In the latter subset, subjects were almost exclusively males and had a relatively advanced age.

**Table 1:** **Number of subjects, percentage of males, mean and standard deviation (sd) of age by occupational category in the whole study and in subjects enrolled in the pretest-posttest study**

**Occupational categories** ^**#**^	**Overall**			**Pretest-posttest study**		
	**Number**	**% Males**	**Age (years) mean ± sd**	**Number**	**% Males**	**Age (years) mean ± sd**
4	1	100.0	44.0			
2	2	50.0	41.5 ± 20.5			
6	3	66.7	53.3 ± 12.9	1	100.0	68.0
12	4	50.0	46.3 ± 9.6			
16	4	25.0	41.3 ± 8.8			
19	4	100.0	45.3 ± 14.2			
21	4	25.0	46.0 ± 4.2			
18	12	41.7	38.3 ± 12.5	1	100.0	61.0
15	18	88.9	34.6 ± 8.4			
11	69	75.4	40.6 ± 8.1	5	100.0	52.4 ± 2.5
3	84	48.8	38.2 ± 9.2	3	100.0	54.3 ± 3.1
13	94	81.9	36.5 ± 8.9	8	100.0	52.0 ± 7.2
14	128	72.7	41.0 ± 9.6	9	100.0	51.3 ± 4.6
7	158	75.3	40.4 ± 8.5	7	100.0	50.1 ± 3.5
1	160	74.4	40.8 ± 9.6	13	84.6	55.2 ± 3.5
9	195	73.3	40.8 ± 9.3	8	100.0	51.5 ± 4.8
23	195	68.2	46.3 ± 8.6	22	100.0	54.0 ± 4.8
10	221	63.8	40.7 ± 9.4	9	100.0	51.6 ± 1.7
20	252	47.6	47.4 ± 7.8	18	94.4	54.6 ± 4.4
5	305	59.0	43.6 ± 8.0	19	100.0	53.4 ± 2.0
17	546	80.2	43.0 ± 9.9	47	100.0	52.8 ± 4.2
8	890	78.5	42.0 ± 8.8	67	98.5	52.3 ± 3.5
22	2187	36.5	42.1 ± 9.2	93	96.8	53.0 ± 3.8
Total	5536	57.5	42.3 ± 9.2	330	97.9	53.0 ± 4.0

^#^Occupational categories: 1 = Food products and beverages (+ Agriculture); 2 = Wood and of products of wood; 3 = Paper and paper products; 4 = Coke and refined petroleum products; 5 = Chemicals and chemical products; 6 = Rubber and plastics products; 7 = Other non-metallic mineral products; 8 = Basic metals; 9 = Machinery and equipment n.e.c.; 10 = Computer, electronic and optical products; 11 = Furniture; 12 = Repair and installation of machinery and equipment; 13 = Electricity, gas, and water supply; 14 = Construction; 15 = Wholesale and retail trade; 16 = Accommodation and food service activities; 17 = Transportation and storage; 18 = Financial and insurance activities; 19 = Real estate activities; 20 = Public administration and defence; 21 = Educaton; 22 = Human health and social work activities; 23 = Other service activities.

Results obtained with the one-group pretest-posttest design are reported in Table 2, showing the proportions with the factor at pretest and posttest, point estimate and confidence interval for the ratio between proportions and exact McNemar significance probability (criterion of positivity in the footnote). It can be seen, in short, that one year after the educational intervention there was a significant increase of physical activity (by 46%; p = 0.0000) and a significant decrease of smoking (by 16%; p = 0.0000), alcohol drinking (by 14%; p = 0.0017), systolic blood pressure (by 17%; p = 0.0009), blood cholesterol (by 15%; p = 0.0004) and cardiovascular risk (by 24%; p = 0.0000).

**Table 2:** **Proportions with the factor (at pretest and posttest), point estimates and confidence intervals (95% CI) for the ratio between proportions and the exact McNemar significance p-value, criterion for positivity in the footnote**

	**Pretest**	**Posttest**	**Ratio post/pre (95% CI)**	**McNemar exact p-value**
	**Proportion with factor**			
Physical activity (PA)	0.42	0.61	1.46 (1.32 - 1.62)	0.0000
Cigarette smoking (CS)	0.40	0.34	0.84 (0.78 - 0.92)	0.0000
Alcohol drinking (AD)	0.45	0.39	0.86 (0.79 - 0.94)	0.0017
Systolic blood pressure (SBP)	0.46	0.38	0.83 (0.74 - 0.92)	0.0009
Blood cholesterol (BC)	0.68	0.58	0.85 (0.78 - 0.93)	0.0004
Cardiovascular risk (CR)	1.00	0.76	0.76 (0.71 - 0.80)	0.0000

Criterion for positivity: physical activity for ≥150 minutes/week (PA); current smoker (CS); regular drinker (AD); systolic blood pressure >140 mmHg (SBP); blood cholesterol >200 mg/dl (BC); cardiovascular risk >5% (CR).

In 108 posttest smokers cigarettes consumption decreased after the intervention; for example, heavy smokers (>20 cigarettes/day) were 43 before and 32 after the intervention. These changes were statistically significant (symmetry exact significance probability = 0.0135).

Table 3 shows the results of the sensitivity analysis. Even assuming that those lost to follow-up kept their pretest value of cardiovascular risk, there was a highly significant difference among pretest and posttest risk of cardiovascular disease (symmetry exact significance probability = 0.0000). In Table 3, the proportions with the factor (CVD risk >5%) were 82.2% (=361/439) at posttest against 100% at pretest. The ratio was 0.82 that indicates a 18% decrease of the cardiovascular risk after the intervention (whereas it was 24% in subjects re-examined at 1 year, Table 2).

**Table 3:** **Sensitivity analysis on the risk of cardiovascular disease: pretest and posttest comparison and exact test of table symmetry in 451 subjects, including 121 lost to follow-up that were presumed to keep the pretest values**

		**Posttest**					
		**<5%**	**5-9%**	**10-14%**	**15-19%**	**>20%**	**Total**
Pretest	<5%	0	0	0	0	0	0
	5-9%	78	269	16	2	1	366
	10-14%	0	22	31	1	2	56
	15-19%	0	3	3	5	0	11
	>20%	0	0	3	0	3	6
	Total	78	294	53	8	6	439

Symmetry exact significance probability = 0.0000.

Table 4 shows the number of subjects in the newly merged occupational categories (work sectors) with the percent of subjects with delta = 1 (pretest cardiovascular risk > posttest cardiovascular risk). The lowest value of delta, indicating the worst impact of intervention, was observed among “basic metals” workers. Values of delta were about twofold higher in other work sectors, suggesting better outcomes. Table 4 also shows ORs with 95% confidence interval (95% CI) and p-value of two models of logistic regression. It can be seen that, after taking into account the changes produced by the intervention (posttest smoking, cholesterol and blood pressure in model 2), the original differences among sectors became no longer significant.

**Table 4:** **Work sectors merged in the analysis of 323 subjects undergoing the pretest-posttest study: number of subjects (N) and percent (%) with delta = 1 (pretest cardiovascular risk > posttest cardiovascular risk), Odds ratio (OR) with 95% confidence interval (95% CI) and p-value for work sectors according to three models of logistic regression (see footnote)**

**Work sectors:**	**Model 1**		**Model 2**	
**N (% with delta = 1)**	**OR**	**p-value**	**OR**	**p-value**
	**(95% CI)**		**(95% CI)**	
Basic metals:	Ref		Ref	
66 (22.7%)	-		-	
Other industries:	2.74	0.008	2.30	0.052
80 (42.5%)	(1.30-5.77)		(0.99-5.31)	
Hospitals:	2.07	0.052	1.56	0.283
90 (35.6%)	(0.99-4.32)		(0.69-3.57)	
Other service activities	2.18	0.040	1.50	0.338
87 (35.2%)	(1.04-4. 59)		(0.65-3.44)	

Model 1: outcome was delta and predictors were age, gender and work sectors.

Model 2: outcome was delta and predictors were age, gender, work sectors, posttest smoking, posttest blood cholesterol and posttest systolic blood pressure.

Table 5 shows the expected number of CVD cases on the basis of cardiovascular risk stratification at pretest and posttest. The total expected cases would be 29 or 23; the difference (6 = 29 – 23) would represent the quota prevented. The relevant cost comprises resources coming from inside the manufacturing process − due to longer interruptions of work during the health surveillance along with the compensation given to OPs for the training received and the time spent in the educational intervention − and external resources acquired from outside the business. Only the latter can be easily quantifiable. They could be about 34,000 euro (14,000 euro for diagnostic kits and 20,000 euro for anti-smoking counseling) that is about 5,700 euro (=34,000/6) per each prevented case or about 10 euro (=14,000/3474) per each examined subject.

**Table 5:** **Expected number of cardiovascular disease cases based on stratification of cardiovascular risk at pretest and posttest**

**Cardiovascular risk %**		**Pretest**		**Posttest**	
**Range**	**Median**	**No. of subjects**	**Expected cases**	**No. of subjects**	**Expected cases**
<5	0.025	0	0	78	2
5-10	0.075	263	20	192	14
10-15	0.125	45	6	41	5
15-20	0.175	10	2	7	1
>20	0.25	5	1	5	1
Total		323	29	323	23

An experienced occupational physician interviewed a small sample of workers already known as “opinion leaders” in their respective groups; the latter reported that both workers and employers perceived the intervention as useful. When the occupational physician was told to express his personal view, he answered: “we have gained esteem of workers”.

Thus, quantitative statistically significant results and some qualitative evidence, together, suggested that the intervention had been effective.

## Discussion

Governmental agencies and private sector groups are working hard to help employers to improve the health of their employees in an efficient, integrated, and cost-effective way. The objective is clear; it is the “how to” that is difficult [2].

At present, the accepted gold standard for the evaluation of interventions in health care is the randomized controlled trial (RCT). The medical literature reports several RCTs on workplace health promotion programs. In a recent meta-analysis, a surprising observation is that studies with poor methodological quality reported an average effect size 2.9-fold larger than good-quality studies. Analyses stratifıed by outcome showed the same result for sickness absence, work productivity, and work ability. This might indicate publication bias: poor-quality studies are more frequently published if they show a greater effect [18]. Another reason could be the fact that RCTs conducted in the worksite may be affected by a threat to internal validity that occurs when the intervention delivered to one group “diffuses” to another (contamination threat). This can easily happen when the intervention is educational in nature, since workers naturally share information with one another. A contamination is undesirable for an evaluation because it reduces the differences observed between the intervention and control groups [15]. Therefore, the logistic requirements of RCTs often cause them to be unfeasible, especially for single smaller worksites.

Given these limitations, the Cochrane Effective Practice and Organization of Care Group endorses three alternative methodologies for evaluating population interventions: (1) the non-RCT, (2) the controlled before-and-after study, and (3) the interrupted time series design. In a non-RCT, individuals or groups are allocated to experimental conditions using a nonrandom method. While nonrandom allocation may be more convenient in some circumstances, it increases the probability that unmeasured characteristics that may influence the outcomes introduces a systematic bias that could artificially exaggerate, or reduce, true intervention effects [15].

It has been recently suggested that the optimal study design for a workplace health promotion program may be a quasi-experimental design in which medical cost data are collected for several years before the program and participants and nonparticipants are matched through propensity scoring [19].

Another approach, whose advantages and methodological limitations have been recently discussed, is multiple baseline design. It involves conducting multiple time-series in multiple populations, each of which receives the intervention at a different point in time [20]. Like RCTs, the multiple baseline design can demonstrate that a change in behavior has occurred, the change is a result of the intervention, and the change is significant. Especially important practical advantages over the RCT are that, first, this design requires fewer population groups and, second, communities may act as their own controls [20]. As explained in Methods, the present study was conducted in multiple occupational categories even though, because of time constraints, we could not stagger the intervention and all categories were examined concurrently.

Individuals examined at posttest was a small fraction of the whole (6% = 323/5,536). This decreased the cost of prevention (about 5,700 euro for each prevented cases of cardiovascular disease) but involved a before-and-after design of the study. The latter is a non-experimental approach that must be used with caution, because of circumstances that threaten the ability to correctly infer whether the intervention had the desired effect. When the basis for choosing the intervention group is a greater apparent need for the intervention, an alternative explanation for the apparent success of the safety initiative is “regression-to the-mean” [15]. In the present study, the intervention included only subjects with a high risk of cardiovascular disease. Thus, part of any decrease observed may have nothing to do with the intervention itself. Rather, CVD risk could be simply fluctuating closer to the average (year-to-year fluctuations). Strictly speaking, however, this consideration applies when one group is being examined and one outcome is being evaluated. As explained in Methods, the present study is a multiple risk factor intervention conducted in multiple occupational categories. There was a consistent performance of all indicators of cardiovascular risk (Table 2). Despite their original heterogeneity, work sectors were not found to influence the posttest risk of cardiovascular disease after taking into account the changes in modifiable risk factors produced by the intervention (Table 4). On the other hand, the characteristics of the intervention group could be altered when enough people drop out of the study (dropout threat) [15]. In the present study the before measurements were available; even in the extreme assumption that all dropouts kept their initial value of cardiovascular risk, a significant decrease of cardiovascular risk was observed at posttest (Table 3). Overall, these pieces of evidence might increase confidence that the intervention was responsible for the change in the outcome.

Cardiovascular risk can be viewed as a surrogate endpoint to investigate the primary event (cause-specific mortality). Adoption of surrogate criteria must, however, be regarded with some caution because the link between surrogate and primary event is not always linear; furthermore risk factor changes could not be maintained in the long term [21].

*The concept of the health promoting workplace is becoming increasingly relevant as more private and public organizations recognize that future success in a globalizing marketplace can only be achieved with a healthy, qualified and motivated workforce … For nations, the development of HPW will be a pre-requisite for sustainable social and economic development* [1]. In this context, health promotion activities fall into the mission of OPs, who should be already trained during the course of education.

We tried to estimate a rough cost of the intervention. Regarding the financial impact of WHP programs, an extensive review of the literature [19] and the major WHP study on cardiac risk factors [22] showed a positive return on investment, demonstrating that such programs seem to pay for themselves.

## Conclusions

The results of this multi-faceted worksite intervention across multiple occupational groups from several geographic communities consistently converged on the evidence of a decreased risk of cardiovascular disease after an educational intervention. The intervention was reasonably priced and easy to integrate within the current organization of occupational health surveillance in Italy.
